# Active fungal infections alter the respiratory microbiome profiles of Mayo Clinic Arizona patients

**DOI:** 10.3389/frmbi.2025.1699912

**Published:** 2025-11-11

**Authors:** Daniel R. Kollath, Kathrine McAulay, Emily A. Higgins Keppler, Kenta S. Reilly, Kenneth K. Sakata, Bridget M. Barker, Thomas E. Grys

**Affiliations:** 1 The School of Sustainable Engineering and the Built Environment, Arizona State University, Tempe, AZ, United States; 2 The Pathogen and Microbiome Institute, Northern Arizona University, Flagstaff, AZ, United States; 3 Deparment of Laboratory Medicine and Pathology, Mayo Clinic Arizona, Phoenix, AZ, United States; 4 Center for Fundamental and Applied Microbiomics, The Biodesign Institute, Arizona State University, Tempe, AZ, United States; 5 Division of Pulmonary Medicine, Mayo Clinic Arizona, Phoenix, AZ, United States

**Keywords:** coccidioidomycosis, candidiasis, lung microbiome, mycobiome, dysbiosis, BALF (Bronchoalveolar lavage fluid)

## Abstract

**Introduction:**

The function of the respiratory microbiome during an active infection is not well characterized. Studies from the gut microbiome suggest a diverse community can aid in modulating the immune system to control infectious pathogens.

**Methods:**

To determine if there are microbial community compositional changes in the human lung during an infection, we conducted an analysis of both the 16S rDNA and the Internal Transcribed Spacer (ITS) region of DNA from bronchoalveolar lavage fluid (BALF) of patients from Mayo Clinic Arizona. In addition to general classification, we assessed differences in the lung microbiome of patients with different infections including coccidioidomycosis, a common fungal pneumonia in Arizona.

**Results:**

We observed patterns of dysbiosis in the lung microbiome during active fungal infection. Patients with active coccidioidomycosis infections had an overabundance of *Malassezia, Epicoccum*, and *Penicillium* species in the fungal communities and bacteria in the classes Bacilli, Bacteroidia, Clostridia, and Gammaproteobacteria. Patients with disseminated coccidioidomycosis showed evidence of extreme dysbiosis in the lung microbiome with a significant overabundance of *Malassezia* and Bacilli. We also observed differences in the fungal communities of patients with an active *Candida albicans* infection, with an overabundance of the genera *Candida* and *Nakaseomyces.* Additionally, we observed a decrease in diversity in the lung fungal communities in patients with an active *Coccidioides* or *Candida* infection but no difference in the bacterial community.

**Discussion:**

These compositional changes in the lung microbiome during an active Coccidioides spp. infection associated with shifts in the fungal community. This is the first study to examine how these fungal pathogens affect the lung microbial community of humans.

## Introduction

### Overview of the lung microbiome

Unlike the gut, oral-cavity, and skin, knowledge of the respiratory microbiome is in its infancy. This is in part due to the historical belief that the lung was a sterile environment, despite its routine contact with the outside world (Laurenzi et al., 1961; Di Simone et al., 2023). Data generated using next generation sequencing technologies are inspiring a rethinking of the presence of microbes in the lung (Dickson and Huffnagle, 2015). To date, most studies of the respiratory environment are observing the “bacteriome” because they only employ 16S sequencing. Other taxa, such as fungi or viruses represent major gaps in knowledge, a deficiency which prohibits a full understanding of the lung microbiome (Di Simone et al., 2023). Studies are also uncovering ample cross-talk between the lung microbiota and lung epithelial cells, and the necessary mechanisms of immune homeostasis (Invernizzi et al., 2020). Increasing availability of data describing the lung microbial community are providing a better understanding of the importance of microbes for respiratory health and disease.

### The role of the lung microbiome in disease

A respiratory microbiome that is in dysbiosis has been associated with increased pathogenesis of certain respiratory pathogens, although the causes are still under investigation (Hilty et al., 2010; Marri et al., 2013; Haspeslagh et al., 2018). This phenomenon has been documented in the gut microbiome but fewer studies that examine the respiratory microbiome exist (Cho and Blaser, 2012; Costea et al., 2018; Sencio et al., 2021). For example, a decrease in overall bacterial diversity with an overabundance of *Streptococcus* and *Prevotella* spp. was associated with greater susceptibility to Influenza A(H3N2) infection (Tsang et al., 2020). The composition of the respiratory microbiome is influenced by three main factors, microbial immigration (inhalation of airborne microbes is the main route), microbial elimination (the clearance of microbes through host immune defenses), and microbial reproduction rate (environmental conditions of the lung microhabitat contribute to high variation) (Gleeson et al., 1997; Dickson and Huffnagle, 2015; Segal et al., 2016; Mathieu et al., 2018; Invernizzi et al., 2020). In active lung disease or infection, the rates of immigration and elimination are unbalanced leading to changes in the respiratory microbiome with some microbes outcompeting others and becoming predominant (Dickson and Huffnagle, 2015). This dysbiosis leads to decreases in species diversity and richness and has been linked to increased mortality and morbidity in chronic respiratory infections (Invernizzi et al., 2020). The vast majority of the above data are from studies of bacterial lung communities, and much less is known about the role of resident fungi in the respiratory tract. However, there is recent evidence that the lung mycobiome (fungal community) plays an important role in clinical outcomes (Knutsen et al., 2012; Denning et al., 2014). For instance, the introduction of the yeast *Candida albicans* to mice that were treated with antibiotics significantly alters the subsequent reassembly of the bacterial communities after the disturbance event which has downstream immune system implications (Erb Downward et al., 2013). Recovery from dysbiosis has been shown to be very important to restoring resilience and health in the gut and is likely important in the lungs (Bloom and Young, 2023). This increasing evidence suggests that commensal resident lung fungi as well as invading fungal lung pathogens can drastically alter the assembly of the respiratory tract microbiome leading to clinical consequences.

### Candidiasis

The relationship between the opportunistic pathogenic yeasts (especially those that were historically in the *Candida* genus) and the bacterial microbiota in the gut, skin and oral cavity have been well studied. For instance, oral inoculation with *Candida albicans* causes severe dysbiosis of the endogenous bacteria in both the oral and small intestine mucosa (Bertolini and Dongari-Bagtzoglou, 2019). Previous work also shows a relationship with the fungal burden of *Candida albicans* and the diversity of the oral microbiome, where increased *Candida* burden leads to a dramatic decrease in bacterial diversity (Kraneveld et al., 2012). Moreover, the authors demonstrate a positive correlation with the bacterial class *Bacilli* and increased *Candida* burden (Kraneveld et al., 2012). Despite investigations of how *C. albicans* impacts on the microbiome, little is known about the role of the mycobiome composition on the respiratory tract. Krause et al. showed that patients admitted to the intensive care unit (ICU) had a shifted lower respiratory tract fungal community that was dominated by *Candida* spp. when compared to healthy controls (Krause et al., 2016). However, the mechanism causing this shift was not clear.

### Coccidioidomycosis

Coccidioidomycosis (Valley fever) is caused by two species of fungal pathogen *Coccidioides immitis* and *C. posadasii* (Fisher et al., 2002). Infection occurs when infectious arthroconidia aerosolize from soil where the fungi are inhabiting and are inhaled by a host. The disease manifests mainly as respiratory infection but can disseminate to other areas of the body such as skin, joints, or the central nervous system (Smith et al., 1948). In endemic and highly populated areas, such as Phoenix and Tucson, Arizona and the San Joaquin Valley in California, up to 30% of community-acquired pneumonia may be attributed to Valley fever infections (Valdivia et al., 2006). Disease phenotypes range from no clinical illness to prolonged disease that can spread beyond the lung to other major organ systems resulting in life-long antifungal therapy, however the mechanisms behind this process remain unknown (Kirkland et al., 2022). A combination of interactions among environmental exposure, pathogen genotype, and host immune response are presumed to play roles (Ward et al., 2021). However, the interaction between *Coccidioides* and the assemblage of the host respiratory microbiome could further contribute to the range in disease severity.

There are few data about the intraspecific interactions between *Coccidioides* spp. and other microorganisms either in a host or in the environment. Certain microbes isolated from the soil have been shown to have inhibitory properties against *Coccidioides* spp. *in vitro* (Lauer et al., 2019; Kollath et al., 2023). Microbes cultured from the trachea and small intestine of mice were shown to inhibit the growth of attenuated *Coccidioides* strains *in vitro* (Tejeda-Garibay et al., 2024). However, these studies do not examine how these microbes contribute to Valley fever disease progression within the respiratory microbiome as a whole. A study examining the fungal lung communities of wild mammals showed that the presence of *Coccidioides* did not correlate to significant changes to the mycobiome; however, *Coccidioides* frequently co-occurred with other potential potentially pathogenic fungi such as *Malassezia, Candida, Blastomyces, and Pneumocystis* species (Salazar-Hamm et al., 2022). To date, there has been no investigation into the role of the respiratory microbiome concurrent with coccidioidomycosis. Therefore, we examined the fungal and bacterial lung communities of patients diagnosed with coccidioidomycosis, as well as other infections and pathologies, at a medical center in the hyper-endemic region of Phoenix, Arizona.

## Methods

### Sample collection and clinical data

Standard-of-care bronchoalveolar lavage (BAL) specimens (n = 102) were collected from individuals presenting to Mayo Clinic Arizona with pulmonary nodules and approved for secondary research use under Mayo Clinic IRB 19-001888. We would like to acknowledge that these samples were collected before the onset of the Covid-19 pandemic. Specimens were stored at -70 °C until extraction. For patients with pulmonary nodules who reside in Arizona, the differential diagnosis is typically cancer, coccidioidomycosis, or mycobacterial infection. So, the intended comparison group were those patients who did not have a lung infection (e.g., cancer or other non-infectious pathologies). Culture, pathology, and other clinical data were abstracted from the patient electronic health record in compliance with Mayo Clinic IRB 19-001888.

### DNA extraction

DNA extraction was performed at the University of Minnesota Genomics Center (UMGC) using the method adapted from that outlined by Davis and colleagues (Davis et al., 2019). Cells from 0.9-1.5 mL BAL fluid were first pelleted by centrifugation at 13,000 x *g* for 5 min at room temperature and resuspended in 90 μL sterile-filtered PBS. Ten microliters Metapolyzyme (Achromopeptidase, chitinase, lyticase, lysostaphin, lysozyme, and mutanolysin; Sigma-Aldrich, Saint Louis, MO) was added to a final concentration of 0.5 mg/mL and the suspension was incubated at 35°C for 16 h. The 100-μL lysate was extracted using the QIAamp BiOstic Bacteremia DNA Kit (QIAGEN, Hilden, Germany) according to the manufacturer’s instructions with one minor modification. After the second CB wash step, a new collection tube was used, and the centrifugation speed was increased to 16,100 x *g*. Negative controls consisted of saline obtained from the Mayo Clinic Arizona bronchoscopy suite and positive controls consisted of 75 μL ZymoBIOMICS Microbial Community Standard (Zymo Research Corporation, Irvine, CA), and ZymoBIOMICS Microbial Community Standard II (Log Distribution) each spiked into 500 μL sterile saline. The community standards contained *Listeria monocytogenes* - 12%, *Pseudomonas aeruginosa* - 12%, *Bacillus subtilis* - 12%, *Escherichia coli* - 12%, *Salmonella enterica* - 12%, *Lactobacillus fermentum* - 12%, *Enterococcus faecalis* - 12%, *Staphylococcus aureus* - 12%, *Saccharomyces cerevisiae* - 2%, and *Cryptococcus neoformans* - 2%. 5 samples yielded pellets that were too big to follow the standard protocol, so for these, 50 uL of the pelleted material was removed to a new tube for resuspension in PBS and addition of the Metapolyzyme.

### DNA amplification and ITS/16S sequencing

Targeted sequencing was also performed at UMGC using previously published primers. Briefly, the V1-V3 region of 16S rRNA gene was targeted using primers 27F: 5`-AGAGTTTGATCMTGGCTCAG-3` and 534R: 5`-ATTACCGCGGCTGCTGG-3` and the fungal ITS region was targeted using 5.8SR: 5`-TCGATGAAGAACGCAGCG-3` and ITS4: 5`-TCCTCCGCTTATTGATATGC-3` (White et al., 1990; Lane, 1991). Paired end (300 bp) sequencing was performed on an Illumina MiSeq using v3 chemistry.

### Microbiome processing and statistical analyses

#### Data processing

Negative saline controls did not amplify any bacterial or fungal taxa. Mock community samples were used to validate the accuracy of the sequencing run. Sequencing of this standard resulted in relative abundances near the theoretical composition with all community members identified in both fungal and bacterial taxa. No bias was detected using the mock communities so these sequences were removed for downstream analysis. ITS2 samples were demultiplexed in Quantitative Insights Into Microbial Ecology 2 using QIIME2v2023.7 (Bolyen et al., 2019) requiring 95% of each read to have a minimum q-score of 20, and allowing no exceptions (-q 19 -r 0 -p 0.95), Forward reads were trimmed of primer sequence, followed by quality control filtering by DADA2 (Callahan et al., 2016) and demultiplexed, trimmed sequences were screened for chimeras using the VSEARCH program (Rognes et al., 2016) and screened for fungal ITS2 sequences with ITSx (Bengtsson-Palme et al., 2013). Operational taxonomic units (OTUs) were assigned taxonomy using BLAST (Van Nguyen and Lavenier, 2009) against the UNITE reference database (Hibbett et al., 2016). The same bioinformatic procedure was done to the 16S sequences however, taxonomy was assigned against the SILVA reference database (Quast et al., 2012). OTU and taxonomy tables were imported into R statistical software program for downstream analyses (Team RC., 2020).

Microbial community data was analyzed using the Phyloseq R package version 3.5.1 (McMurdie and Holmes, 2013). Fungal data was rarefied to the lowest depth sample (2111 reads), and the bacterial data was rarefied to the lowest depth sample (11756 reads). OTU richness (Chao1 index), observed Alpha diversity and Shannon diversity index was used to calculate alpha diversity. To statistically examine differences in Alpha diversity a Wilcoxon rank-sum non-parametric test was used to compare two groups and a Kruskal-Wallis non-parametric test was conducted to compare more than two groups followed by a *post hoc* Dunn’s test for pairwise comparisons. Bray-Curtis distance matrix was used to examine for beta diversity between the treatment groups. Differences in beta diversity was evaluated with PERMANOVA followed by *post-hoc* pairwise PERMANOVA test using the Bray-Curtis dissimilarity distance matrix. To visualize the dissimilarity of microbial communities between treatment groups principal coordinate analysis (PCoA), ordination plots were created. Indicator species analysis was conducted using the R indicspecies package which considers the associations of species based on general abundance, presence-absence, and individual bases and calculates a correlation value (Cáceres and Legendre, 2009).

#### Data availability

Sequences are publicly available at NCBI under the project number PRJNA1309155.

## Results

### Community composition

The microbiome of bronchoalveolar lavage (BAL) fluid was examined from a total of 102 patients. Eighty of the patients did not have a formal coccidioidomycosis diagnosis at the time of sample collection. Twelve patients in the cohort had a previous diagnosis of coccidioidomycosis, but were not exhibiting active disease at the time of BAL collection; these patients were classified in the analysis as not having active coccidioidomycosis. Twenty-two patients had active coccidioidomycosis at the time of BAL collection, and two patients had disseminated coccidioidomycosis at the time of BAL collection. Fifteen patients in the cohort had *Candida albicans* positive cultures with no positive test for coccidioidomycosis at the time of sample collection.

**Figure 1:**
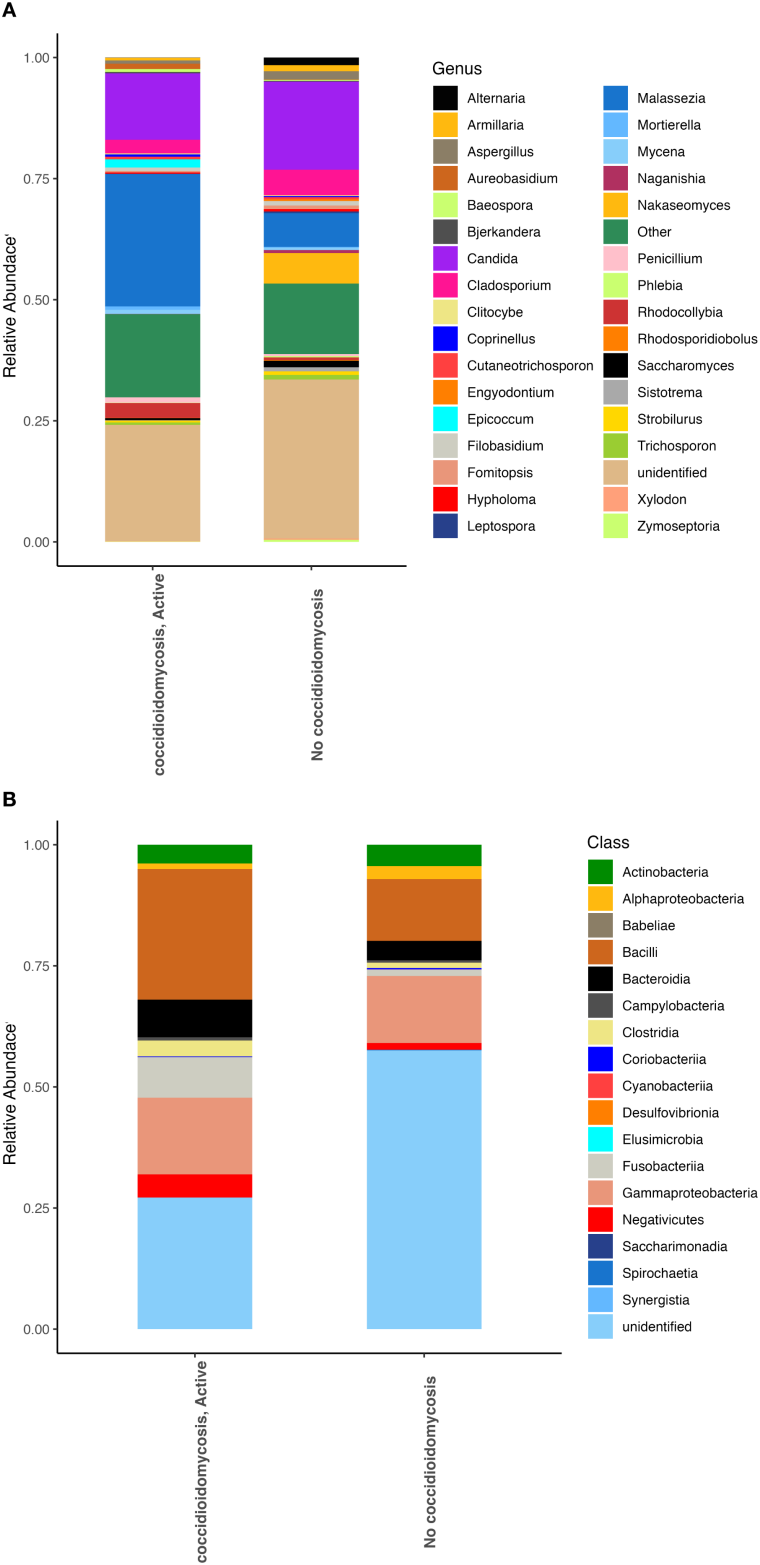
Fungal and bacterial community composition varies based on *Coccidioides* infection. Relative abundances were calculated to represent the proportion of each fungal and bacterial taxa in relation to the whole community. **(A)** Fungal community structure at the genus level. **(B)** Bacterial community structure at the class level.

We examined compositional differences in both the fungal and bacterial communities between patient groups. Patients with active coccidioidomycosis showed higher relative abundances of the fungal genera *Malassezia, Epicoccum*, and *Penicillium* (**Figure 1A**). Patients with no active coccidioidomycosis infection had significantly greater abundance of species from the genera *Candida*, *Cladosporium*, and *Nakaseomyces* (**Figure 1A**). Interestingly, the two patients with disseminated coccidioidomycosis had significantly greater species from the genus *Malassezia* when compared to respiratory disease and no disease (**Supplementary Figure S1a-b**). When the two disseminated patient samples were compared to each other, the fungal community composition was quite similar (**Supplementary Figure S1b**). For bacteria, patients with an active coccidioidomycosis infection had greater relative abundances of the bacteria from the classes Bacilli, Bacteroidia, Clostridia, and Gammaproteobacteria when compared to patients with no active *Coccidioides* infection (**Figure 1B**). Patients with disseminated coccidioidomycosis had a significantly greater relative abundance of bacteria in the class Bacilli when compared to patients with no coccidiomycosis diagnoses or respiratory disease (**Supplementary Figure S1c**). However, when the two disseminated patient samples were compared to each other, the bacterial communities were very different, with one patient contributing to the significant increase in Bacilli (**Supplementary Figure S1d**). This is evidence that disseminated coccidioidomycosis may alter the fungal lung communities in a signature way while the alteration in the bacterial communities is unpredictable or may have multiple associated dysbiotic states.

An indicator species analysis was conducted to examine if there were taxa that were significantly upregulated in certain patient groups (**Table 1**). The fungal species *Tausonia pullulans*, *Botrytis* spp., and *Mortierella alpina* were all found to be associated with active respiratory coccidioidomycosis. Two species from the genus *Malassezia*, species from the genus *Penicillium, Hortaea werneckii, Ceriporia xylostromatoides, Preussia terricola*, and *Candida glaebosa* were found to be associated with disseminated coccidioidomycosis, acknowledging this is a limited set (**Table 1**). No bacterial association with disease was found with this indicator species analysis. Several other clinically important fungi were detected in the BAL samples from the patient cohort with Next Generation Sequencing (NSG) analysis. A list of these species can be found in **Table 2**.

**Table 1:** Most significant taxa that were identified at least to genus by means of an indicator species analysis.

Species	Group	P-value
*Tausonia pullulans*	Active, Respiratory Coccidioidomycosis	0.044
*Botrytis* spp.	Active, Respiratory Coccidioidomycosis	0.045
*Mortierella alpina*	Active, Respiratory Coccidioidomycosis	0.044
*Malassezia* spp.	Disseminated Coccidioidomycosis	0.001
*Malassezia arunalokei*	Disseminated Coccidioidomycosis	0.009
*Hortaea werneckii*	Disseminated Coccidioidomycosis	0.02
*Penicillium* spp.	Disseminated Coccidioidomycosis	0.02
*Candida glaebosa*	Disseminated Coccidioidomycosis	0.03
*Ceriporia xylostromatoides*	Disseminated Coccidioidomycosis	0.03
*Preussia terricola*	Disseminated Coccidioidomycosis	0.02

**Table 2:** Medically important fungi detected with ITS2 Illumina sequencing in patient BAL.

Candida spp.	Malassezia spp.	Other spp. of interest
*Candida albicans*	*Malassezia restricta*	*Fusarium oxysporum*
*Candida tropicalis*	*Malassezia globosa*	*Cryptococcus neoformans*
*Candida parapsilosis*	*Malassezia arunalokei*	*Pneumocystis jirovecii*
*Candida sake*	*Malassezia sympodialis*	*Fusarium solani*
*Candida glabrata*	*Malassezia japonica*	*Alternaria infectoria*
*Candida dubliniensis*	*Malassezia slooffiae*	
*Candida palmioleophila*		
*Candida glaebosa*		
*Candida sorbosivorans*		

**Figure 2:**
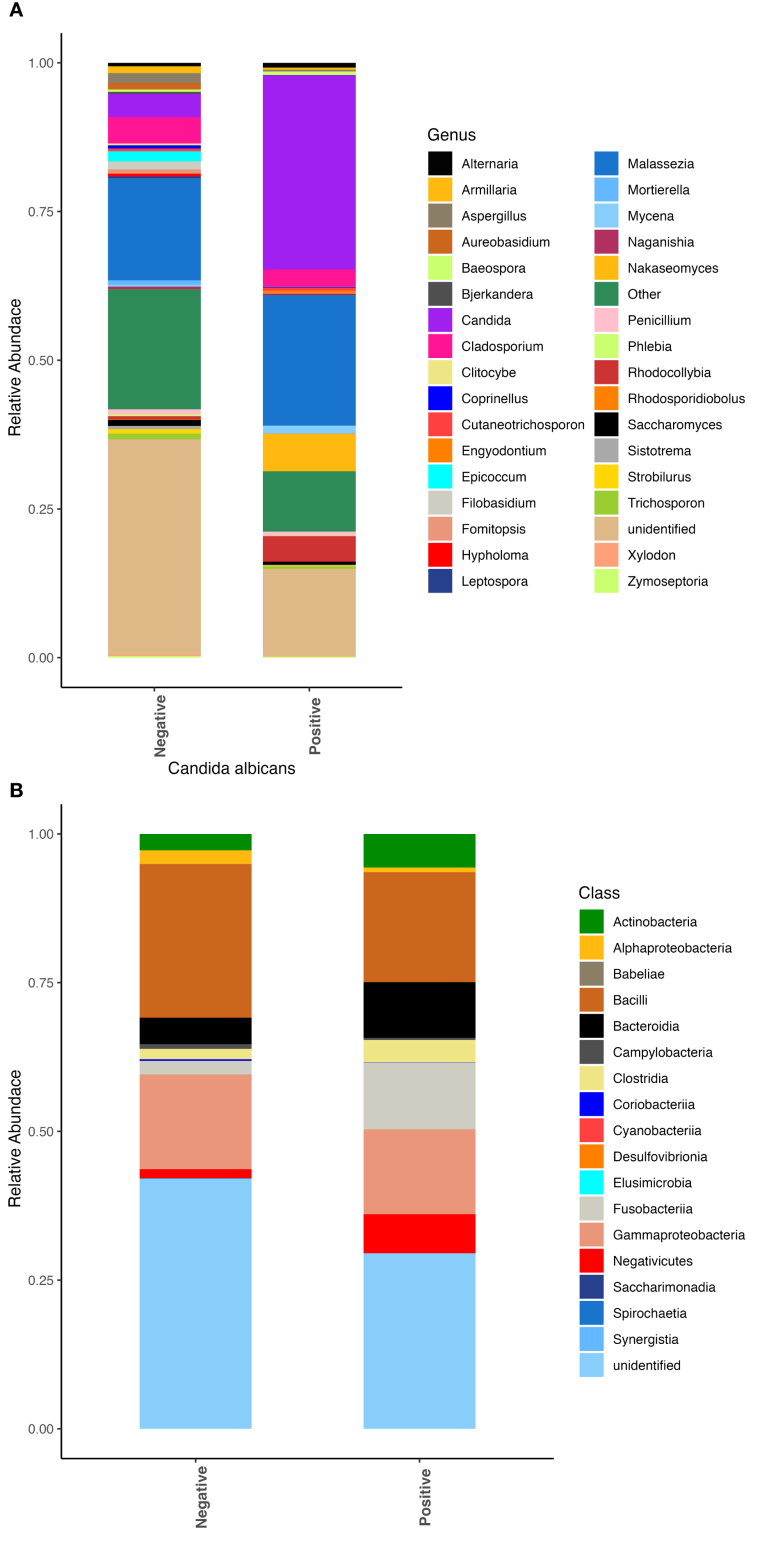
Fungal and bacterial community composition varies based on *Candida albicans* infection. Relative abundances were calculated to represent the proportion of each fungal and bacterial taxa in relation to the whole community. **(A)** Fungal community structure at the genus level. **(B)** Bacterial community structure at the class level.

We also observed differences in the respiratory microbial communities in patients who had an active *Candida albicans* infection, as shown with a positive fungal culture, compared to patients for whom *C. albicans* was not recovered on culture. Patients with active *C. albicans* infection had significantly greater abundances of the fungi from the genera *Candida* and *Nakaseomyces* (**Figure 2A**). Few bacterial differences were observed besides slight increases in abundance of the classes Bacteroidia and Fusobacteria (**Figure 2B**).

### Alpha diversity

**Figure 3:**
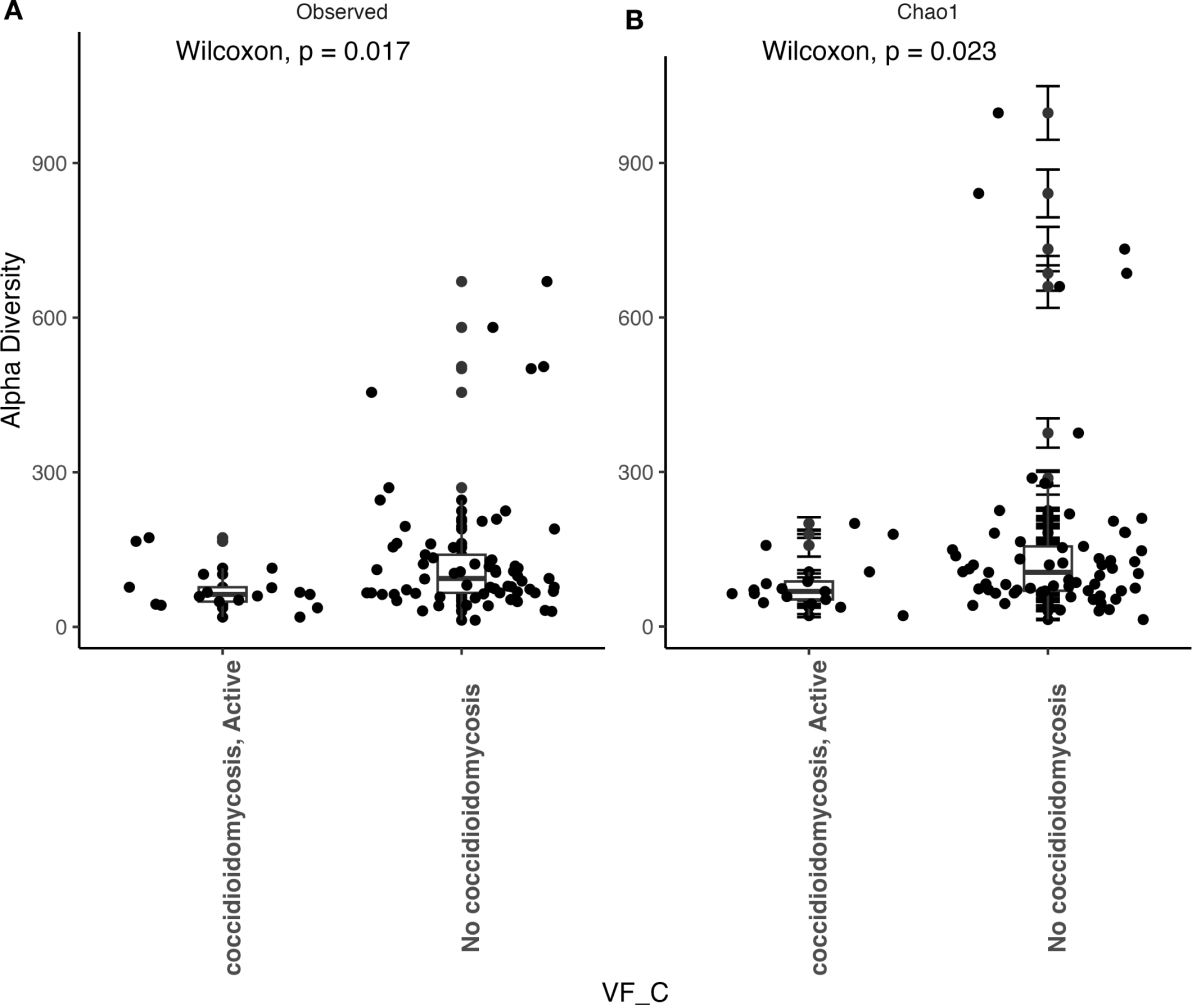
Fungal alpha diversity is significantly reduced during an active *Coccidioides* infection. **(A)** Comparison of observed alpha diversity between active coccidioidomycosis patients and non-infected patients, expressed as median and range values. **(B)** Comparison of estimated species richness with the Chao1 index, expressed as median and range values. Error bars on the outlier points are there to indicate the standard error defined by the Chao l model for estimating species richness.

**Figure 4:**
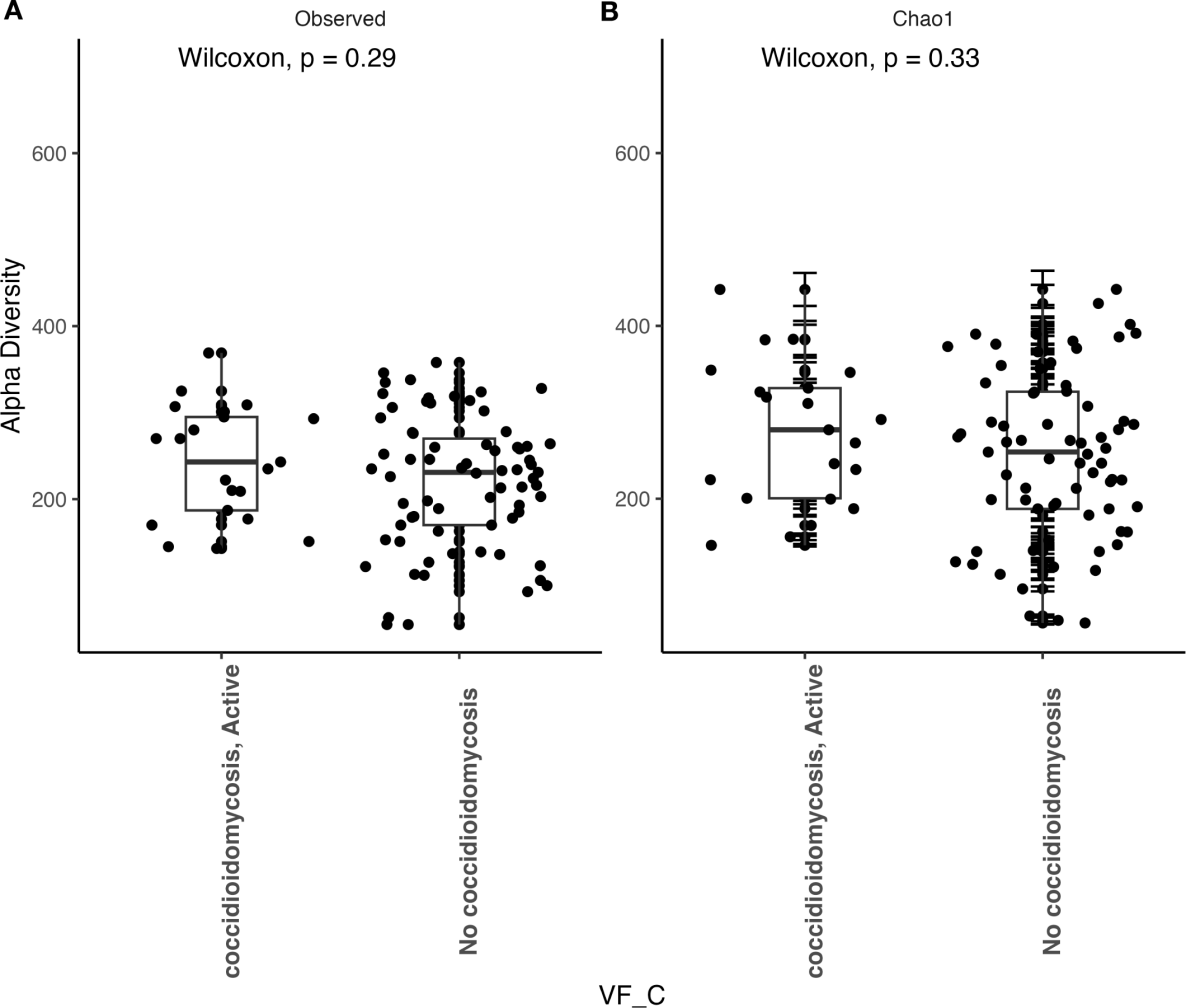
Bacterial alpha diversity does not change during an active *Coccidioides* infection. **(A)** Comparison of observed alpha diversity between active coccidioidomycosis patients and non-infected patients, expressed as median and range values. **(B)** Comparison of estimated species richness with the Chao1 index, expressed as median and range values. Error bars on the outlier points are there to indicate the standard error defined by the Chao1 model for estimating species richness.

**Figure 5:**
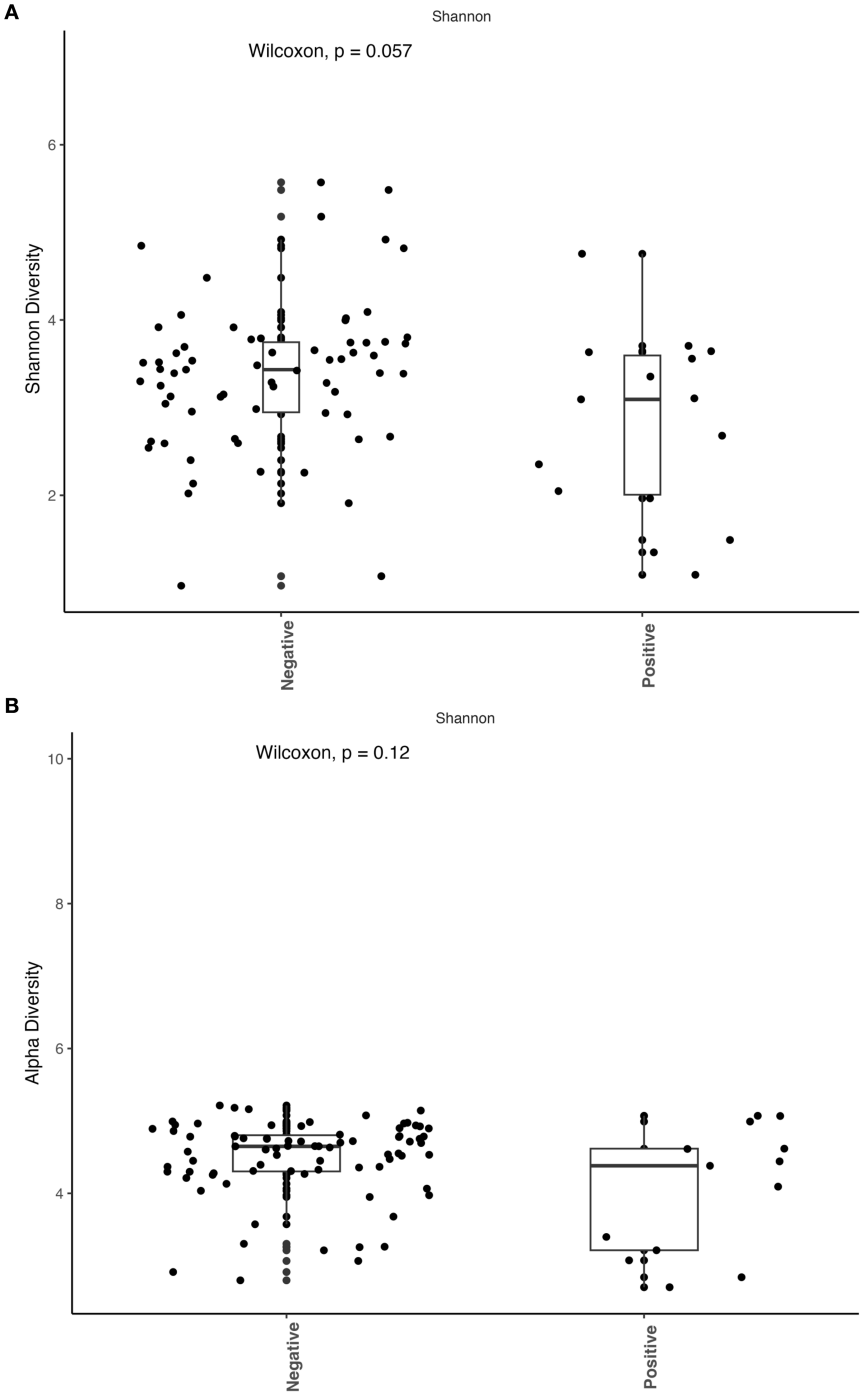
Active *Candida albicans* infection slightly decreases alpha diversity of the fungal community but does not affect the bacterial community. The Shannon index was used to estimate alpha diversity between patients with active C albicans infection and non-infected patients. **(A)** Comparison of observed fungal alpha diversity, expressed as median and range values. **(B)** Comparison of observed bacterial alpha diversity, expressed as median and range values.

We observed that patients with active coccidioidomycosis had significantly reduced fungal alpha diversity (p=0.017) and species richness (p=0.023) when compared to patients with no active coccidioidomycosis infection (**Figure 3**). We did observe a slight decrease in fungal alpha diversity and species richness in patients with disseminated disease, although this was not statistically significant (**Supplementary Figure S2**). This may be due to the small sample size of disseminated patients. We did not observe any differences in bacterial alpha diversity or species richness between patients with active disease and non-active disease (**Figure 4**) or in patients with disseminated disease (**Supplementary Figure S3**). Bacterial alpha diversity does not appear to be influenced by *Coccidioides* spp. infection; however, the fungal community appears to be susceptible to perturbation that is associated with the pathogen. We observed a slight decrease in Shannon Diversity in patients with active *C. albicans* infection (**Figure 5A**; p=0.057) and no difference in the bacterial community (**Figure 5B**; p=0.12). This is another indication that the respiratory fungal community is more susceptible to disruption due to an invading fungal pathogen.

### Beta diversity

**Figure 6:**
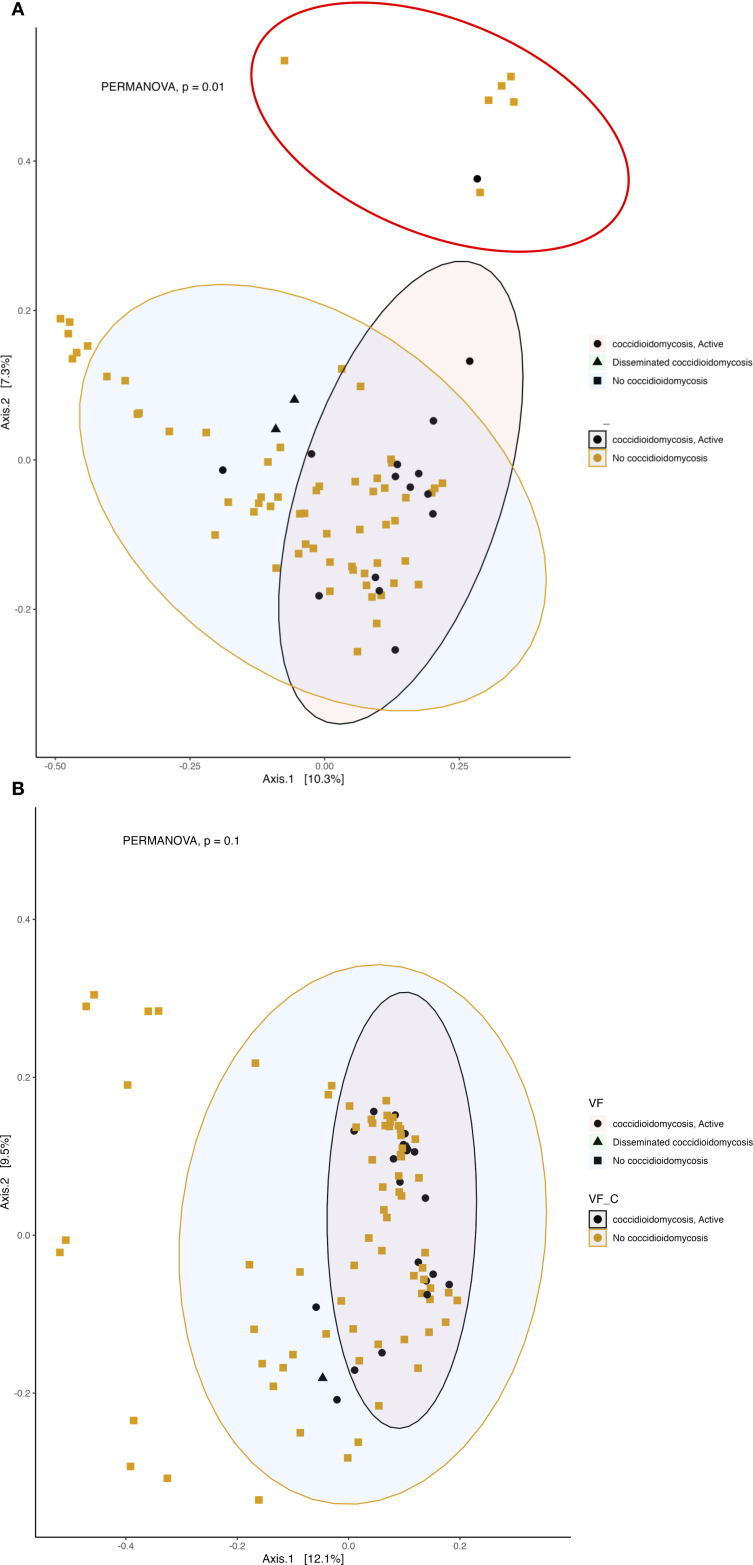
Active fungal infection influences the beta diversity of respiratory fungal communities but has little effect on the bacterial community. Principal coordinates analysis (PCoA) of Bray-Curtis dissimilarity. 95% confidence ellipses are shown for each group. Colors represent active infection and no infection and shapes represent no infection, respiratory or disseminated infection. **(A)** Beta diversity of fungal community. The red circle represents a distinct cluster of patients with active *albicans* infection. **(B)** Beta diversity of bacterial community.

We observed a correlation between fungal community composition and coccidioidomycosis infection status (PERMANOVA p=0.01). Patients experiencing active coccidioidomycosis infection had distinct fungal profiles when compared to patients with no active infection at the time of sample collection (**Figure 6A**). Patients with disseminated infections also appear to exhibit distinct fungal profiles and cluster together; however, pairwise PERMANOVA analysis is not statistically different when compared to no infection and respiratory infection (**Figure 6A**; p=0.28, p=0.27, respectively). The statistical power is quite low when comparing the disseminated patients due to the small sample size. Interestingly, we do observe a distinct cluster that is driven by active *C. albicans* infection (shown in the red circle), and PERMANOVA analysis confirms that this association is statistically significant (p=0.001). We observed no difference in beta diversity of the bacterial community composition based on coccidioidomycosis infection status (**Figure 6B**; PERMANOVA, p=0.1). We do observe a small amount of clustering in the PCoA; however, these patients have a mixture of bacterial, fungal, and viral infections with no clear association on what is driving this clustering. *C. albicans* active infection does not influence the bacterial community composition (PERMANOVA, p=0.08).

## Discussion

The literature regarding the influence of pathogen invasion on host microbial composition (Altmäe et al., 2019; Longford et al., 2019) led us to ask how the presence of fungal pathogens might change the respiratory fungal and bacterial compositions in patients with varying disease states. To our knowledge this is the first investigation in human coccidioidomycosis patients on the influence of the fungus on the lung microbiome. Our principal finding from this investigation is that active infection with *Coccidioides* or *Candida albicans* significantly changes the respiratory fungal communities of patients in Arizona. The role of the mycobiome during infection is mixed. Historically, abiotic factors, such as pH and temperature, were thought to be the main drivers of the mycobiome; however, these studies lack an assessment of species co-occurrences influencing the fungal community composition (Mishra et al., 2021). Recently, that thinking has changed but the majority of studies have been focused on patients with altered immune responses, such as HIV, cystic fibrosis, irritable bowel syndrome and Crohn’s Disease (Ott et al., 2008; Qin et al., 2010; Hoarau et al., 2016; Mishra et al., 2021). As far as we are aware, this is the first evidence of two fungal pathogens correlated with the respiratory mycobiome of humans.

Interestingly, we did not find an association between active infection and the bacterial community composition. Several studies, in various animal systems, have shown a disruption in the bacteriome associated with active fungal infection. In a laboratory-controlled setting, the fungal opportunistic pathogen *C. albicans* significantly altered the bacterial community reassembly after antibiotic disturbance (Erb Downward et al., 2013). In amphibians, the infection history of the fungal pathogen *Batrachochytrium dendrobatidis* significantly altered the skin microbiome community structure making animals more susceptible to severe disease (Bates et al., 2022). This evidence shows how a fungal pathogen can cause the normal host microbiota to shift. The lack of an association in the current study between infection and the bacteriome could be due to the health status of the patient cohort, as many had co-morbidities and medication use.

Numerous studies have looked at the importance of a microbiome in symbiosis (healthy) and the potential role in preventing severe infection (such as dissemination). This is done through a variety of mechanisms such as direct inhibition of the pathogen via niche competition and the promotion and activation of barrier immunity via microbial associated molecular patterns (MAMPs) of the commensal microbes (Reid et al., 2001; Bartlett, 2002; Petersson et al., 2011; Khosravi and Mazmanian, 2013). However, these data were mostly collected in the context of response of the gut bacteriome to a bacterial pathogen, such as *Clostridium difficile* (Loo et al., 2005; Brandt et al., 2012). Little is known about the role fungi play in disseminated disease. Furthermore, little is known about the mechanisms that lead to disseminated coccidioidomycosis outside of the lung. Our results may provide clues as to the role that a diverse respiratory fungal community has on limiting the risk of severe coccidioidomycosis. Although robust conclusions cannot be drawn based on the small sample size of disseminated patients.

In patients with active and disseminated disease we detected core taxa associated with these disease states with indicator species analysis. *Tausonia pullulans, Botrytis* spp., and *Mortierella alpina* were fungi associated with active disease. *Malassezia arunalokei* and *Candida glaebosa* were the most notable fungal species associated with disseminated disease. An influx of *Malassezia* spp. in many different parts of the body has been shown in people with chronic disease and immunocompromised patients and could be and indicator of certain chronic conditions (Abdillah and Ranque, 2021). This could be a reason we were able to detect a large number of OTUs in patients with disseminated coccidioidomycosis and is an indicator of an overall decrease in health. Malassezia are however associated with the oral cavity and skin and contamination during bronchoscopy could have occurred. However, results remain unchanged after accounting for sampling bias with fungal mock community controls. Rickettsiales spp. bacterial were also found to be associated with disseminated disease. This is a diverse bacterial order that includes important intracellular pathogens (Giannotti et al., 2022); however, we cannot determine if these bacterium are indicators of severe coccidioidomycosis. Other fungi of medical importance were detected across all patient populations. The most notable are several species in the *Candida* and *Malassezia* genera, *Cryptococcus neoformans*, and *Pneumocystis jirovecii.* but we cannot determine if these fungi are resident members of the lung community or causing disease from an observational study. They could be indicators of the overall health of this patient population as patients with disseminated disease show decreased alpha diversity and overabundance of certain taxa (signs of a microbiome in dysbiosis); however, the low sample size (n=2) makes it difficult to draw significant conclusions.

The results obtained from this study are a function of the patient population where the samples were collected and have limitations. All patients were from Mayo Clinic Arizona and no patients from outside of the endemic area for Valley fever were used as controls. An inherent limitation to the study design is that no BAL were from healthy patients, i.e. patients without symptoms or radiological findings. BAL collection is invasive and would not be conducted on patients without a clinical indication to undergo the procedure. Patients who were negative for coccidioidomycosis at the time of sample collection may have been exposed to the pathogen in the past. Serologic status was not always available, and seropositivity does not definitively correlate with presence of active or previous infection. The majority of patients had several co-morbidities that may play a role in the alteration of the microbiome. Several of the patients were on several different medications (including anti-fungal and antibiotics) at the time of sample collection. To address this, analyses were done to examine the contribution of anti-microbials to the alteration of the lung microbiome and no such significance was detected.

In summary, this study provides novel insights into the impact of fungal pathogens on the respiratory microbiome of patients with coccidioidomycosis, highlighting a significant shift in fungal community composition associated with active and disseminated disease. While no clear association was found between fungal infection and bacterial community changes, the presence of specific fungal taxa suggests that fungal dysbiosis may be a marker of disease severity and overall health status. These findings underscore the importance of considering the mycobiome in respiratory disease research, particularly in endemic regions like Arizona. Although limitations such as small sample size and lack of healthy controls constrain broader generalizations, this work lays the foundation for future investigations into the role of fungal communities in disease progression and immune modulation. Understanding these microbial dynamics may ultimately inform strategies to mitigate severe outcomes in fungal respiratory infections.

## Data Availability

The datasets presented in this study can be found in online repositories. The names of the repository/repositories and accession number(s) can be found in the article/**Supplementary Material**.

## Supplementary material

Supplementary Figure 1 Fungal and bacterial community composition vary based on disseminated *Coccidioides* infection. Relative abundances were calculated to represent the proportion of each fungal and bacterial taxa in relation to the whole community. A) Fungal community structure at the genus level. B) Fungal community structure at the genus level of the two patient samples with disseminated CM. C) Bacterial community structure at the class level. D) Bacterial community structure at the class level of the two patient samples with disseminated CM.

Supplementary Figure 2 Fungal alpha diversity is slightly reduced during disseminated *Coccidioides* infection. A) Comparison of observed alpha diversity between active coccidioidomycosis patients and non-infected patients and disseminated infection, expressed as median and range values. B) Comparison of estimated species richness with the Chao1 index, expressed as median and range values.

Supplementary Figure 3 Bacterial alpha diversity is not influenced during disseminated *Coccidioides* infection. A) Comparison of observed alpha diversity between active coccidioidomycosis patients and non-infected patients and disseminated infection, expressed as median and range values. B) Comparison of estimated species richness with the Chao1 index, expressed as median and range values.
