# Supplementary figures and images for: Active fungal infections alter the respiratory microbiome profiles of Mayo Clinic Arizona patients
Source: Front Microbiomes. 2025 Nov 11;4:1699912. doi: 10.3389/frmbi.2025.1699912 (PMC12993690; doi:10.3389/frmbi.2025.1699912)

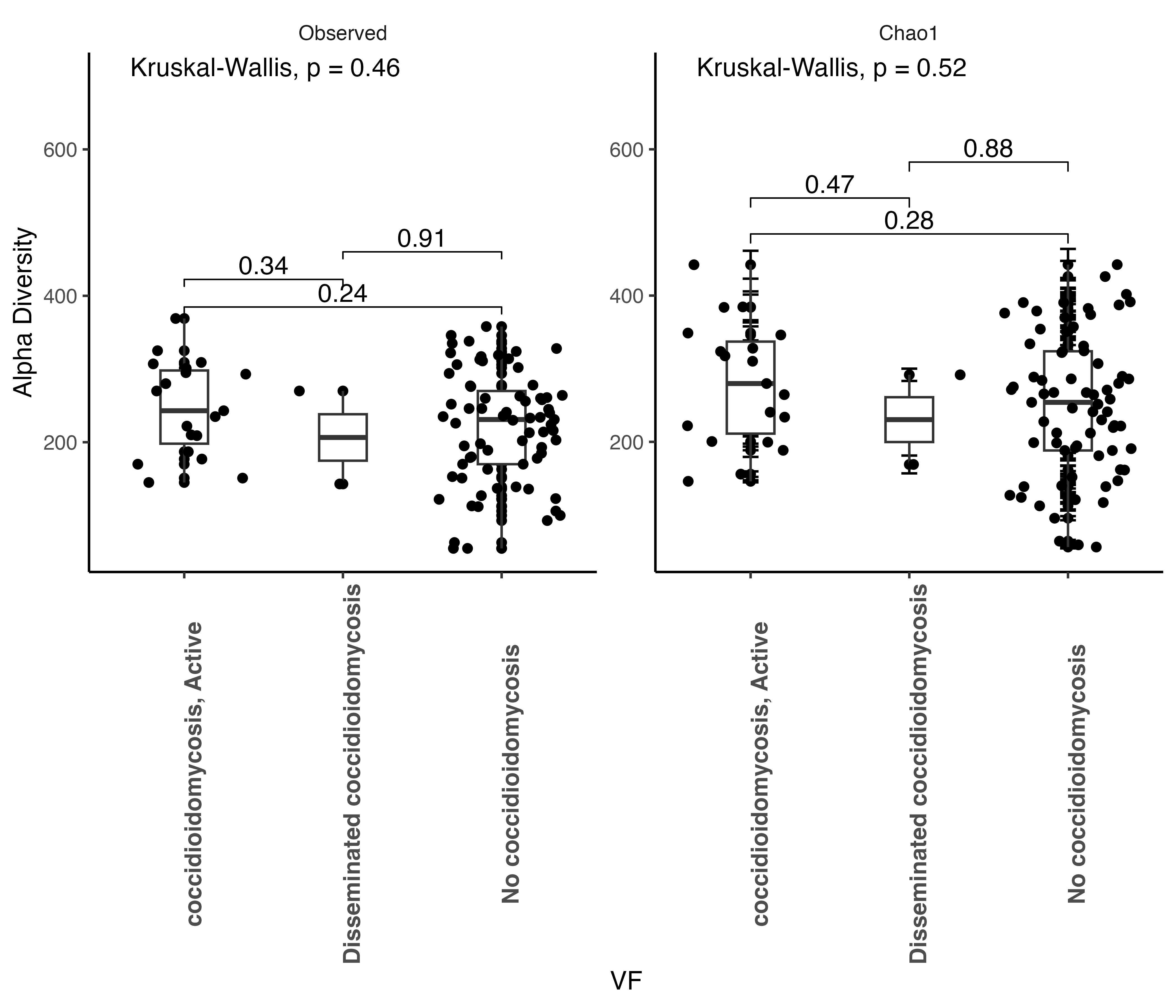

Supplement: Supplementary Figure 1 — Fungal and bacterial community composition vary based on disseminated Coccidioides infection. Relative abundances were calculated to represent the proportion of each fungal and bacterial taxa in relation to the whole community. A) Fungal community structure at the genus level. B) Fungal community structure at the genus level of the two patient samples with disseminated CM. C) Bacterial community structure at the class level. D) Bacterial community structure at the class level of the two patient samples with disseminated CM. [file Supplementaryfile1.zip › Supplemental figure 3.TIFF]

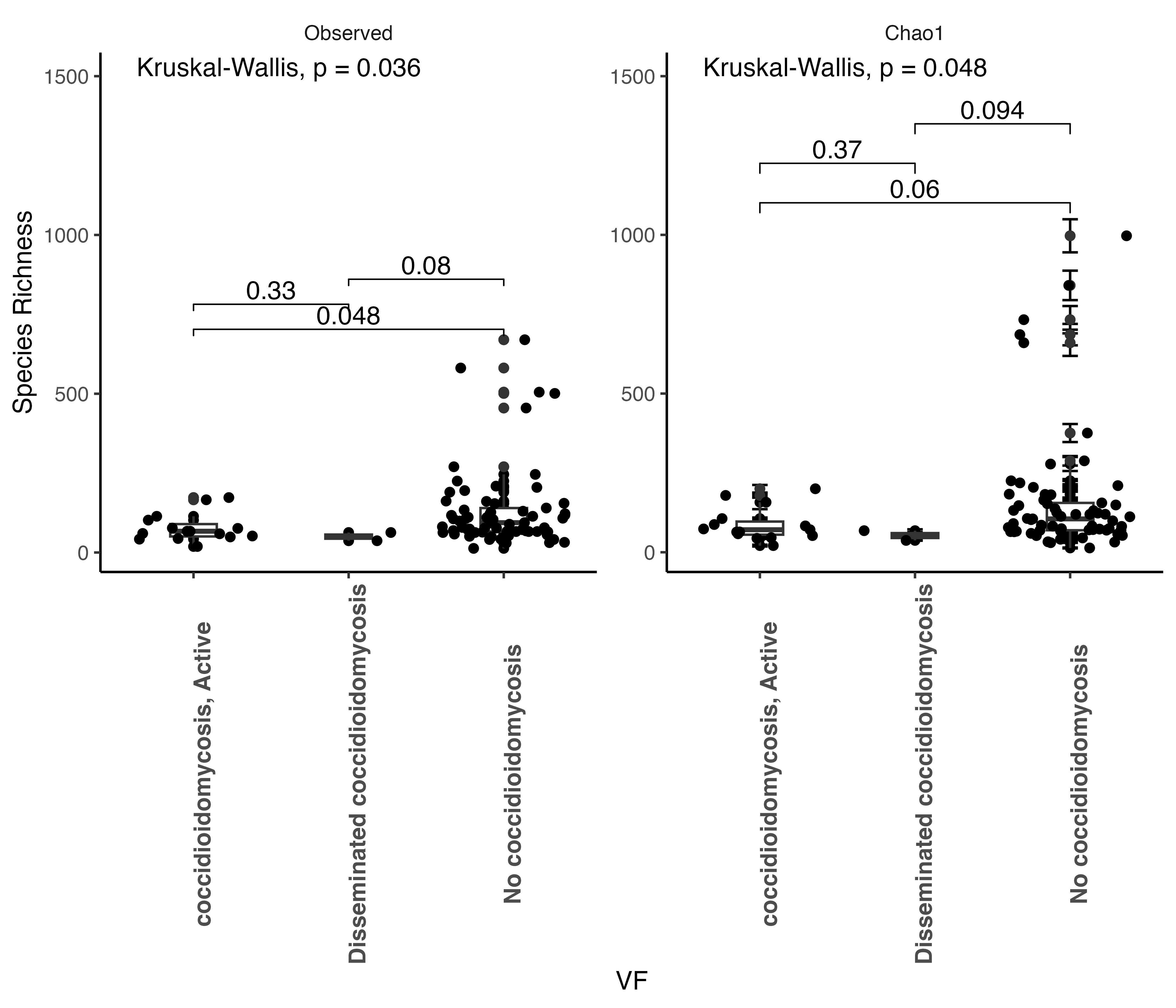

Supplement: Supplementary Figure 1 — Fungal and bacterial community composition vary based on disseminated Coccidioides infection. Relative abundances were calculated to represent the proportion of each fungal and bacterial taxa in relation to the whole community. A) Fungal community structure at the genus level. B) Fungal community structure at the genus level of the two patient samples with disseminated CM. C) Bacterial community structure at the class level. D) Bacterial community structure at the class level of the two patient samples with disseminated CM. [file Supplementaryfile1.zip › Supplemental figure 2.TIFF]

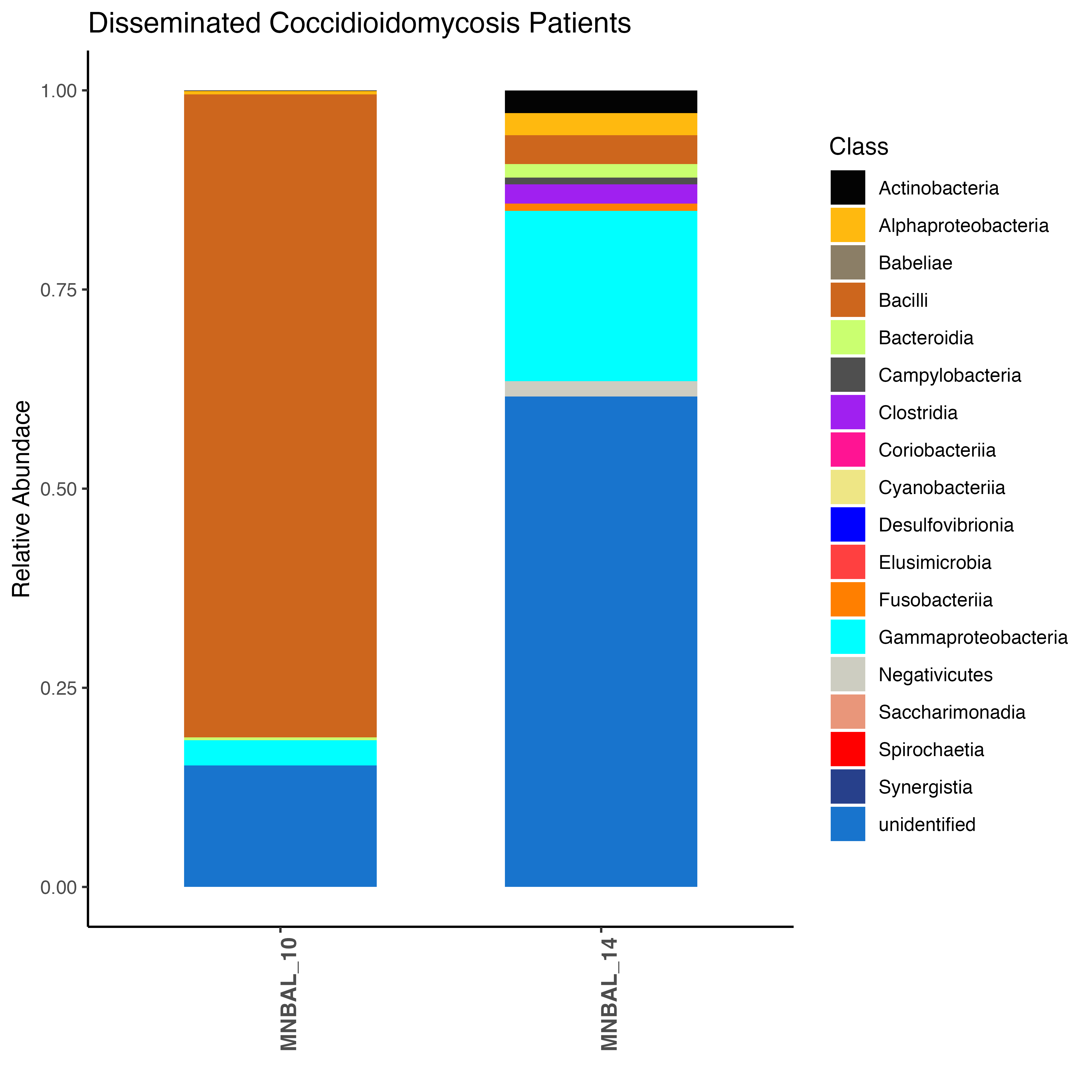

Supplement: Supplementary Figure 1 — Fungal and bacterial community composition vary based on disseminated Coccidioides infection. Relative abundances were calculated to represent the proportion of each fungal and bacterial taxa in relation to the whole community. A) Fungal community structure at the genus level. B) Fungal community structure at the genus level of the two patient samples with disseminated CM. C) Bacterial community structure at the class level. D) Bacterial community structure at the class level of the two patient samples with disseminated CM. [file Supplementaryfile1.zip › Supplemental figure 1d.TIFF]

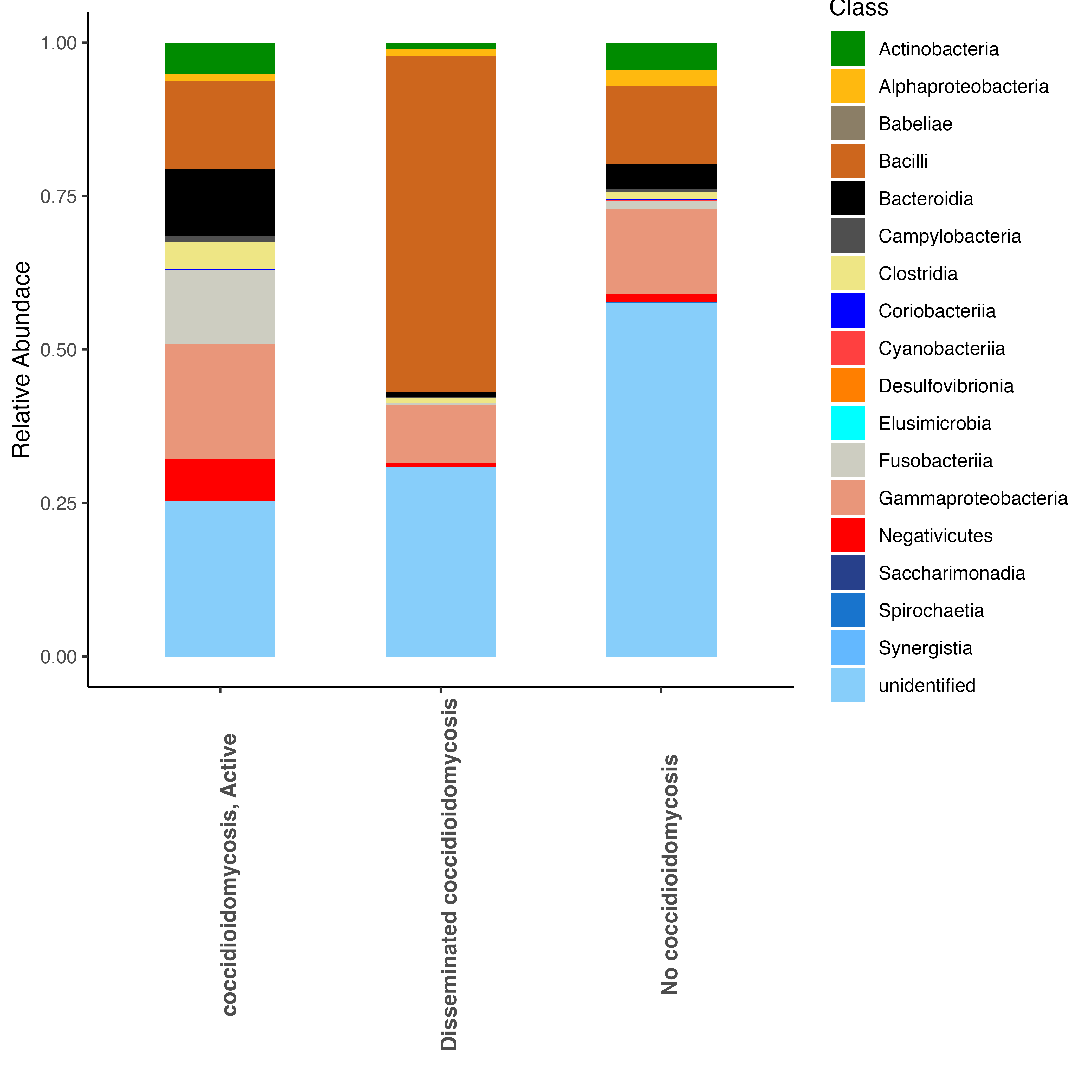

Supplement: Supplementary Figure 1 — Fungal and bacterial community composition vary based on disseminated Coccidioides infection. Relative abundances were calculated to represent the proportion of each fungal and bacterial taxa in relation to the whole community. A) Fungal community structure at the genus level. B) Fungal community structure at the genus level of the two patient samples with disseminated CM. C) Bacterial community structure at the class level. D) Bacterial community structure at the class level of the two patient samples with disseminated CM. [file Supplementaryfile1.zip › Supplemental figure 1c.TIFF]

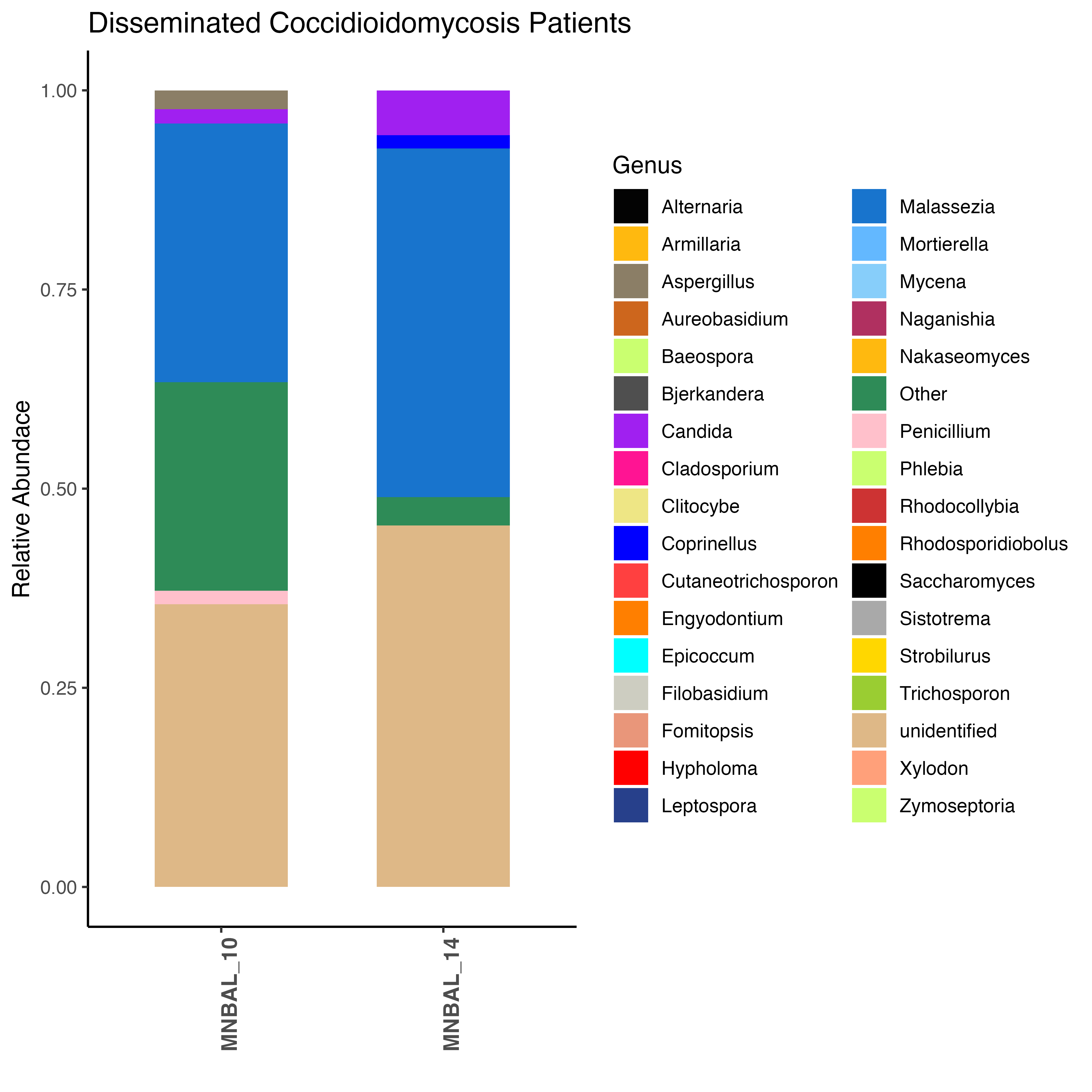

Supplement: Supplementary Figure 1 — Fungal and bacterial community composition vary based on disseminated Coccidioides infection. Relative abundances were calculated to represent the proportion of each fungal and bacterial taxa in relation to the whole community. A) Fungal community structure at the genus level. B) Fungal community structure at the genus level of the two patient samples with disseminated CM. C) Bacterial community structure at the class level. D) Bacterial community structure at the class level of the two patient samples with disseminated CM. [file Supplementaryfile1.zip › Supplemental figure1b.TIFF]

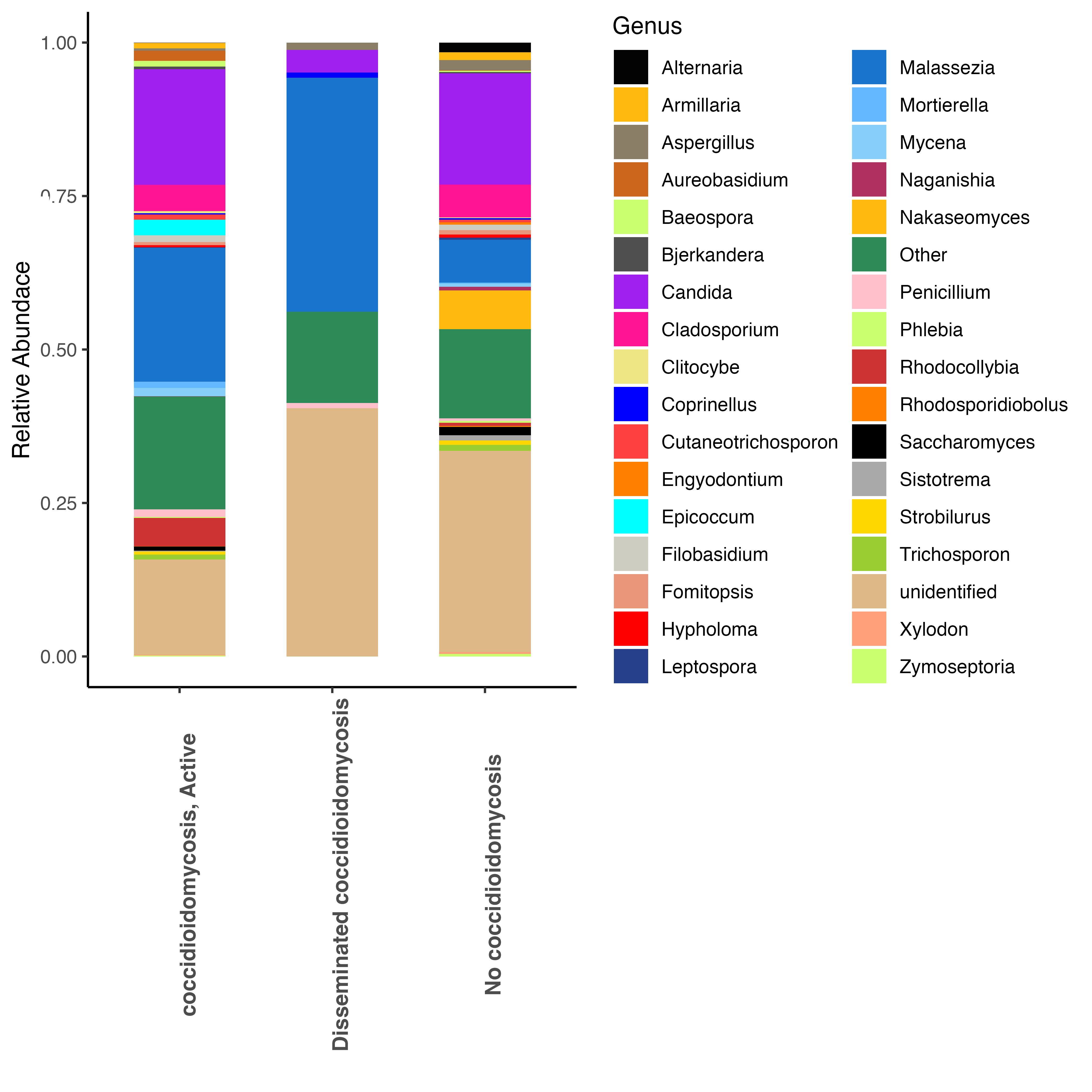

Supplement: Supplementary Figure 1 — Fungal and bacterial community composition vary based on disseminated Coccidioides infection. Relative abundances were calculated to represent the proportion of each fungal and bacterial taxa in relation to the whole community. A) Fungal community structure at the genus level. B) Fungal community structure at the genus level of the two patient samples with disseminated CM. C) Bacterial community structure at the class level. D) Bacterial community structure at the class level of the two patient samples with disseminated CM. [file Supplementaryfile1.zip › Supplemental figure 1a.TIFF]
